# Coenzyme Q10 alleviates neurological deficits in a mouse model of intracerebral hemorrhage by reducing inflammation and apoptosis

**DOI:** 10.3389/ebm.2025.10321

**Published:** 2025-02-28

**Authors:** Xiaoqing Yang, Yi Zhao, Sisi Yu, Lihui Chi, Yeyan Cai

**Affiliations:** ^1^ Department of Neurosurgery, Ruian People’s Hospital, The Third Affiliated Hospital of Wenzhou Medical University, Wenzhou, Zhejiang, China; ^2^ Department of Traditional Chinese Medicine, Ruian Tangxia People’s Hospital, Wenzhou, Zhejiang, China

**Keywords:** intracerebral hemorrhage, coenzyme Q10, autologous blood injection, inflammation, apoptosis

## Abstract

This research study was directed towards to assessing whether coenzyme Q10 (CoQ10) is linked to neuroprotection and induces anti-inflammatory and anti-neuronal death responses in an Intracerebral hemorrhage (ICH) mouse model via right caudate nucleus injection with collagenase VII. Autologous blood was injected into mice to induce ICH. We found that FoxM1 was upregulated in the ICH-injured animals. Moreover, CoQ10 treatment effectively ameliorated neurological deficits, mitigated cerebral edema, and minimized hematoma in model mice, demonstrating dose-dependent efficacy and promoting the functional recovery of the animals. ELISA and real-time PCR assays of pro-inflammatory cytokines indicated that CoQ10 was capable of alleviating neuroinflammation in ICH. In line with the part of CoQ10 in attenuating the inflammatory response, CoQ10 also suppressed cell apoptosis in the ICH-injured brain, which partly accounts for its neuroprotective effect. Furthermore, our analysis of different inflammatory pathways indicated that CoQ10 targeted the nuclear factor-kappa B signaling axis. Our findings suggest that CoQ10 protects against ICH by mitigating neuroinflammatory responses and preventing neuronal apoptosis, with the underlying mechanism possibly being connected with nuclear factor-kappa B pathway regulation. Therefore, CoQ10 holds significant potential as a therapeutic strategy for treating ICH.

## Impact statement

This study verified the function of CoQ10 in protecting against brain injury caused by ICH and that its neuroprotective effect is in part due to its inhibition of pro-inflammatory cytokine secretion and neuronal death. Moreover, CoQ10 was confirmed to suppress pro-inflammatory cytokine secretion by inhibiting p65 phosphorylation.

## Introduction

Intracerebral hemorrhage (ICH), including the rupture of cerebral blood vessel and blood leakage into the cerebral parenchyma [1, 2], takes up 10%–15% in stroke cases and exhibits a high incidence and mortality [3]. Cerebral impairment resulting from ICH occurs in two stages. The initial bleeding destroys the brain cell structure while the hematoma induces elevated intracranial pressure, thereby affecting blood circulation and causing brain herniation [4]. The second stage, which can be avoided, will continue for several hours or days [5, 6] and includes local inflammation [7], the generation of clotting components, and impairment of the perihematomal tissues (such as the blood–brain barrier) [8]. Some proofs indicate that secondary injury may be triggered by the generation of thrombin, hemoglobin, and iron [9–12]. Therefore, efficient treatment of hemorrhagic stroke-induced secondary impairments is warranted.

Ubiquinone, also known as CoQ10, is a kind of lipophilic, vitamin-like compound which plays a pivotal role in the mitochondrial electron transport system and contributing to ATP biosynthesis [13]. It is generally acknowledged that CoQ10 exhibits potent antioxidant effects and also enhances defensive effects of other antioxidative enzymes [14]. Aside from protecting neuronal cells via its antioxidative activity, CoQ10 has demonstrated the ability to improve brain-derived neurotrophic factor (BDNF) levels and enhance its signaling pathway in the brain, which accounts for its neuroprotective effects [15]. The effects of CoQ10 have been proven in numerous neurological diseases [16, 17]. Meanwhile, several reports have investigated the neuroprotective impacts of CoQ10 in a variety of stroke models, such as symptomatic vasospasm-triggered ischemic brain lesions [18]. Moreover, CoQ10 administration has been shown to ease venous ischemia/reperfusion injuries [19]. However, studies on the neuroprotective role of CoQ10 during ICH development and pathogenesis are scarce. Therefore, this research was designed to explore the neuroprotective impacts of CoQ10 against neurological deficits caused by ICH damage and the underlying molecular mechanism.

## Materials and methods

### Laboratory animals

Male C57BL/6 mice, with a weight range of 18.0–20.0 g, were acquired from Vital River Biotechnology Co., Ltd., China. All animal-related experiment operations had obtained the approval of Animal Care and Use Committee of Ruian People’s Hospital, the Third Affiliated Hospital of Wenzhou Medical University, in full accordance with the ARRIVE guidelines. Mice were raised in individual cages under a 12-h light and dark cycle in controlled temperature conditions, and provided sufficient food and water.

### Experimental grouping

To examine the impacts of CoQ10 administration in an animal model of ICH, 36 C57 mice were arbitrarily allocated to six groups, consisting of a sham group (n = 6) and five ICH subgroups (n = 30, successful ICH mode number). The five subgroups that had been subjected to ICH injury were as follows (each n = 6): ICH group (no treatment), ICH + vehicle group (intragastric administration with phosphate-buffered saline at day 0 post ICH), ICH + lCoQ10 group (intragastric administration with low-dose CoQ10, 0.5 mg/g mouse weight at day 0 post ICH), ICH + mCoQ10 group (intragastric administration with medium-dose CoQ10, 5 mg/g mouse weight at day 0 post ICH), and ICH + hCoQ10 group (intragastric administration with high-dose CoQ10, 25 mg/g mouse weight at day 0 post ICH).

One mouse from each of the six groups was decapitated on day 1 following assessments of neurological function deficits caused by ICH. Subsequently, on day 5, one mouse from each of the six groups was used for neurological function deficit assessments, whereas the remaining mice were decapitated. Cerebral specimens were harvested from the sacrificed animals for biochemical examination.

### ICH model establishment

Following established protocols [20], the mice were given sodium pentobarbital for anesthesia and subsequently positioned in a stereotaxic frame. ICH model establishment was implemented in accordance with a previously described method [21]. In brief, through a 1 mm burr hole, infusion of a solution containing 0.5 U of collagenase VII was given to the right caudate nucleus at a rate of 0.4 μL/min, with the stereotaxic coordinates set at 1.0 mm posterior to the pons, 3.0 mm to the right, and 6.0 mm ventral to the skull. The sham group was subjected to identical operations except for the intracerebral injection. Upon awakening from the anesthesia, the mice were raised in cages and furnished sufficient water and food.

### RNA isolation and quantitative PCR analysis

Cerebral specimens, kept at −80°C, were processed to isolate total RNA using TRIzol reagent as the relevant instructions. The synthesis of cDNA was carried out utilizing a PrimeScript RT Master Mix (Takara, Dalian, China) as the relevant guidelines.

qRT-PCR (model 7500 Real-Time PCR System; Applied Biosystems) was implemented utilizing the SYBR Green kit (Takara, Dalian, China) as the relevant guidelines. The glyceraldehyde 3-phosphate dehydrogenase (GAPDH) gene served as a reference for data normalization. To quantify the expression of target mRNA relative to GAPDH in each sample, the cycle threshold (Ct) was utilized in the equation: expression = 2e−ΔCt, with ΔCt representing the deference between Ct_target_ and Ct_GAPDH_. The sequences of primers applied in qPCR were detailed below:

**Table:** 

Genes	Forward	Reverse
IL-1β	5′-CCA CAG ACC TTC CAG GAG AAT G-3′	5′-GTG CAG TTC AGT GAT CGT ACA GG-3′
IL-6	5′-AGA CAG CCA CTC ACC TCT TCA G-3′	5′-TTC TGC CAG TGC CTC TTT GCT G-3′
TNF-α	5′-CTC TTC TGC CTG CTG CAC TTT G-3′	5′-ATG GGC TAC AGG CTT GTC ACT C-3′
GAPDH	5′-GCA CCG TCA AGG CTG AGA A-3′	5′-TGG TGA AGA CGC CAG TGG A-3′
Bcl-2	5′-CAT TTC CAC GTC AAC AGA ATT G-3′	5′-AGC ACA GGA TTG GAT ATT CCA T-3′
Bax	5′-AGC TGA GCG AGT GTC TCA AG-3′	5′-GTC CAA TGT CCA GCC CAT GA-3′

### Neurological deficit tests and determination of brain tissue water content

Corner and limb placement tests, components of neurological deficit tests (NDTs), were performed on day 5 after ICH induction, using previously described methods [22]. The NDTs were conducted by a technician unaware of mouse grouping.

An electronic balance was employed for measuring ipsilateral cerebral hemisphere wet weight. The brains were dried at 100°C for 1 day, after which their dry weight was determined. The formula applied for water content calculation: (wet weight – dry weight)/wet weight × 100%.

### Determination of neurological severity score

The mice underwent evaluation utilizing the modified neurological severity score (mNSS) test [23] which is similar to human contralateral neglect test and includes balance, movement, reflex, and sensory evaluations. The results were graded on a point scale of 0–18.

### Hematoxylin and eosin staining

Transcardial perfusion of the mice with saline, followed by 4% paraformaldehyde, was conducted 5 days after inducing ICH. Subsequently, the brain was separated, post-fixed overnight, immersed in sucrose solutions (15% and 30%, 1 day each) at 4°C, and then frozen. Slices of the frozen tissue, each 8 μm thick, were prepared. Hematoxylin and eosin (H&E) staining was implemented as the relevant guidelines.

### ELISA

The pro-inflammatory cytokines (IL-1beta, IL-6, and TNF-alpha) levels in culture supernatants and cerebral tissue homogenates (for 10 mg cerebral tissue, add 1 mL of ice-cold lysis buffer and homogenize using electric homogenizer and stored in −80°C) were measured utilizing ELISA kits (BMS224-2, EH2IL6, and BMS2034, Invitrogen) in accordance with the manufacturer guidelines.

### TUNEL assay

The TUNEL assay was utilized to assess cell death. In brief, the frozen brain tissue was defrosted at ambient temperature and underwent a 0.5-h fixation using 4%paraformaldehyde. Next, the slides were soaked for 5 min in Triton X-100 (0.1%) and subjected to 60-min incubation at 37°C using TUNEL reaction mixture.

### Western blot assay

Whole-cell lysates were obtained utilizing RIPA buffer (pH 8.0) added protease inhibitor cocktail, and their protein concentrations were determined utilizing BCA kit. Using SDS-PAGE, the proteins were separated and subsequently transferred to PVDF membranes. After the vacant sites on each membrane had been blocked, it was subjected to overnight incubation at 4°C utilizing primary antibodies. Then, following a wash with Tween-containing Tris-buffered saline (TBST), the membranes were exposed to secondary antibodies for 1-h incubation at room temperature. Finally, after rinsing with TBST several times, the protein bands were revealed using a Maximum Sensitivity Substrate Kit (Thermo Fisher Scientific, Waltham, MA, United States). The information for used antibodies (all from Abcam) is displayed here: Caspase-3 antibody (1:2000, ab4051), cleaved Caspase-3 antibody (1:500, ab2302), Caspase-9 antibody (1:2000, ab25758), cleaved Caspase-9 antibody (1:500, ab2324), Bax antibody (1:5000, ab53154), Bcl-2 antibody (1:5000, ab59348), GAPDH antibody (1:5000, ab8245), p-NF-kappaB antibody (1:1000, ab16502), p-STAT3 antibody (1:500, ab30647), p-p38 antibody (1:500, ab4822), p-p52 antibody (1:1000, ab227078).

### Statistical analysis

The data are reported in the format of the mean ± SD. The distinctions among different groups and between two groups were examined utilizing one-way ANOVA and t-test, respectively. P < 0.05 meant noticeable differences.

## Results

### CoQ10 administration restored neurological functions and reduced the brain water content in the ICH-injured animals

**FIGURE 1:**
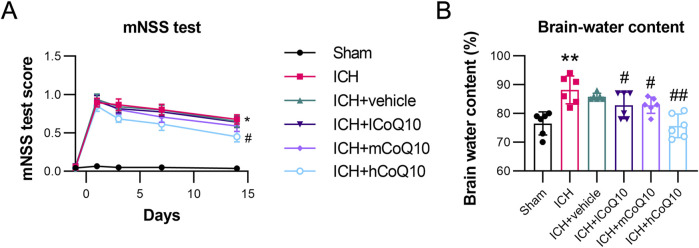
Neurobehavioral test results and brain water content of intracerebral hemorrhage (ICH)-injured mice. **(A)** The mNSS test of CoQ10-treated mice was conducted on days −1, 1, 3, 7, and 14 after ICH induction. **(B)** Brain water content was determined at day 5 after intracerebral hemorrhage induction. *P < 0.05, **P < 0.01 vs. Sham; ^#^P < 0.05, ^##^P < 0.01 vs. ICH.

To verify the functional outcome of CoQ10-mediated neuroprotection in ICH, the mice in the ICH groups were exposed to different doses of CoQ10, and a series of neurobehavioral tests were performed. Neurological alterations were detected 1 day prior to ICH induction and at intervals of 1, 3, 7, and 14 days thereafter. CoQ10 (0.5, 5, and 25 mg/g) was injected 60 min following ICH induction. The high-dose CoQ10 group demonstrated lower mNSSs compared with the ICH + vehicle group at 7 and 14 days following ICH induction (Figure 1A). To assess the role of CoQ10 in brain lesions in the ICH-injured mice, water content in the brain was measured on day 5 after injury induction. In contrast to the sham group, the ICH model group demonstrated elevated brain water contents. However, medium and high doses of CoQ10 decreased the brain water contents in the ICH-injured mice (Figure 1B), attenuating the brain edema in a dose-dependent manner.

### CoQ10 treatment attenuated neurological function deficits and brain impairment in ICH-injured mice

**FIGURE 2:**
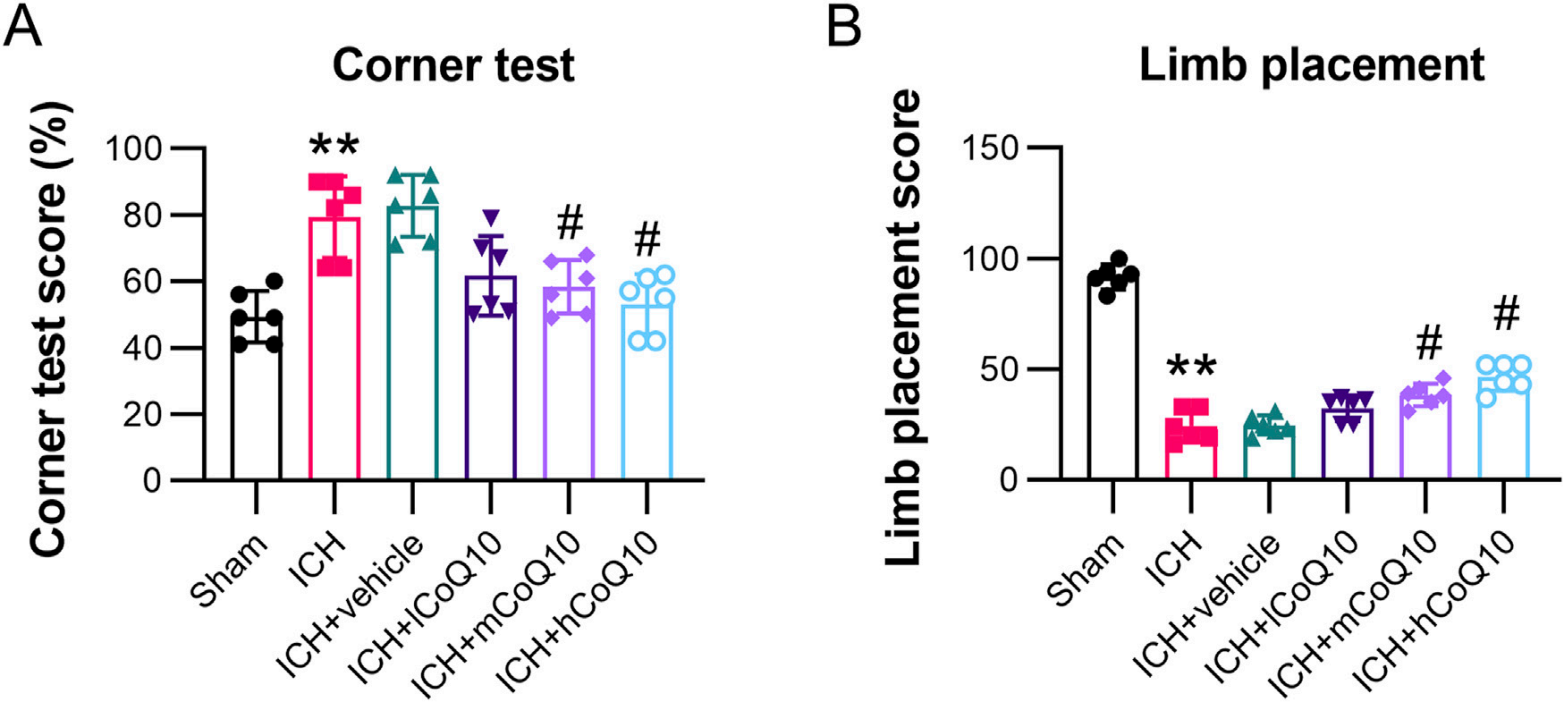
Administration of CoQ10 improved neurological function and eased cerebral impairment in intracerebral hemorrhage-injured mice. **(A, B)** Corner and forelimb placement tests were conducted at day 5 following intracerebral hemorrhage induction. **P < 0.01 vs. Sham; ^#^P < 0.05 vs. ICH.

Behavioral assessments were performed 5 days after ICH induction. The ICH group exhibited markedly elevated right-turn frequencies and decreased limb placement scores in contrast to the sham group results. By contrast, the different doses of CoQ10 restored the impaired neurological functions, especially at the high dose (Figures 2A, B).

**FIGURE 3:**
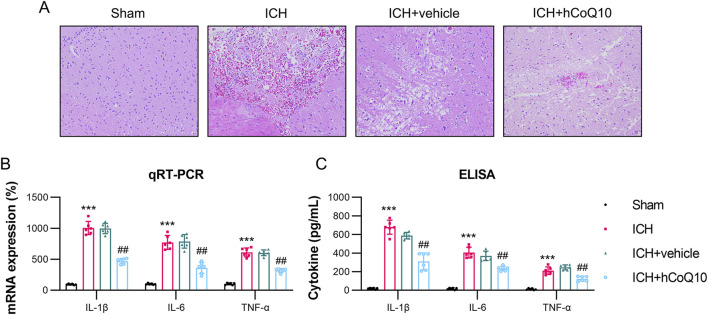
CoQ10 treatment attenuated pathological changes and neuroinflammation in intracerebral hemorrhage-injured mice. **(A)** H&E staining of the hematoma area at day 5 following intracerebral hemorrhage induction. **(B)** ELISA results of pro-inflammatory cytokine expression levels homogenate of the brain tissue. **(C)** qPCR results of pro-inflammatory cytokine mRNA expression in the homogenate of hematoma-affected brain tissue. ***P < 0.001 vs. Sham; ^##^P < 0.01 vs. ICH.

Furthermore, according to the findings of H&E staining of the hematoma, histological differences existed between the ICH and sham groups. However, in the high-dose CoQ10-treated mice, the hematoma area was reduced than that in the ICH + vehicle mice (Figure 3A). The findings indicated that CoQ10 administration could ameliorate neurological dysfunction and brain impairment in ICH-injured mice.

### CoQ10 reduced the expression of pro-inflammatory cytokines in ICH-injured mice

To monitor the impact of CoQ10 on neuroinflammation in the ICH-injured mice, qPCR and ELISA were performed to examine the pro-inflammatory cytokine levels in the homogenate and supernatant of the brain tissue. As indicated by their increased levels in the tissue homogenate, ICH modeling markedly promoted the expression of cytokines, such as IL-1β, IL-6, and TNF-α, whereas treatment with CoQ10 reduced the expression of these three cytokines (Figure 3B). Furthermore, the ELISA data confirmed that the administration of CoQ10 could alleviate the inflammatory response in the ICH-injured mice (Figure 3C).

### CoQ10 reduced the death of brain cells in ICH-injured mice

**FIGURE 4:**
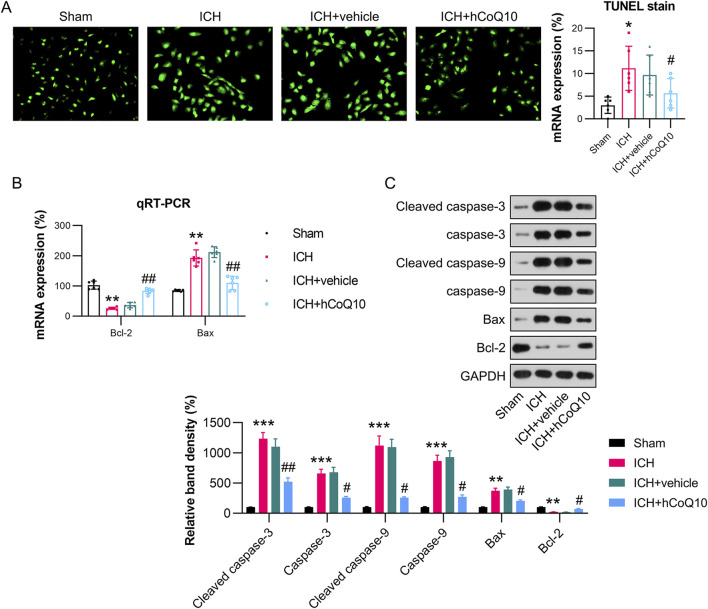
CoQ10 treatment inhibited neuronal apoptosis in intracerebral hemorrhage-injured mice. **(A)** TUNEL assay detection of apoptotic cells. **(B)** qPCR results of Bcl-2 and Bax mRNA expression levels in hematoma-affected brain tissue. **(C)** Western blot results of Bcl-2, Bax, cleaved caspase-3, and caspase-9 levels. *P < 0.05, **P < 0.01 vs. Sham; ^#^P < 0.05, ^##^P < 0.01 vs. ICH.

The levels of brain cell apoptosis in the ICH-injured mice were assessed utilizing TUNEL, qPCR, and western blot (WB) assays. Among them, TUNEL assay revealed that the ICH model group exhibited an elevated number of apoptotic cells, whereas the CoQ10-treated group showed a reduction (Figure 4A). The qPCR finding revealed that the mRNA level of Bcl-2 was reduced, whereas that of Bax was elevated, after ICH induction. However, CoQ10 treatment reversed these Bcl-2 and Bax levels (Figure 4B). WB analysis of expression of Bax, Bcl-2, cleaved caspase-3, and caspase-9 further confirmed that CoQ10 treatment reduced apoptosis in the perihematomal tissue of the ICH-injured animals (Figure 4C). These results indicate that CoQ10 alleviates ICH-induced neuronal cell apoptosis and neuroinflammation *in vivo*.

### CoQ10 suppressed p65 phosphorylation in the hematoma area of brain tissue in ICH-injured mice

**FIGURE 5:**
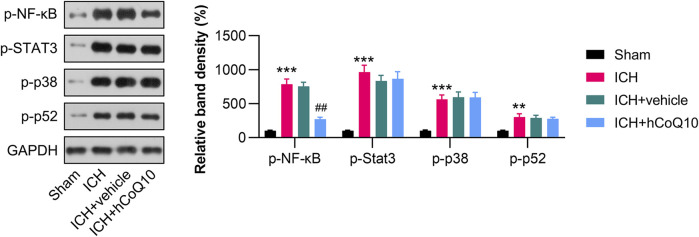
CoQ10 treatment inhibited the phosphorylation of p65 in the hematoma area of ICH-injured mice. Western blot results of the NF-κB, STAT3, p38, and p52 protein levels in the perihematomal brain area and in PCNs.

Since NF-κB, STAT3, p38, and p52 sensors are responsible for inflammation, we examined whether these molecules are regulated by CoQ10. The western blot findings indicated that NF-κB, STAT3, p38, and p52 phosphorylation was promoted in hematoma-affected tissue in response to ICH injury (Figure 5). CoQ10 treatment markedly reduced the phosphorylation of p65, but not that of STAT3, p38, and p52, suggesting that the neuroprotective function of CoQ10 is attributed to NF-κB deactivation.

## Discussion

ICH, a prevalent and destructive brain disorder, has higher incidence and mortality compared with those of ischemic stroke [24]. ICH injury can trigger cell death signaling, thereby causing a new stage of inflammation. The immune system is stimulated by a set of signals released by dead cells [25]. Although several investigations have demonstrated the neuroprotective impacts of CoQ10 on ICH [26], its definitive activity in protecting against this type of hemorrhagic injury remains unverified. This study is the first to confirm that CoQ10 can mitigate neuroinflammation and neuronal cell death in the perihematomal region as well as ameliorate neurological dysfunction following ICH injury. The results revealed that the inhibition of both pro-inflammatory cytokine secretion and neuronal death was associated with the neuroprotective effects of CoQ10 following experimental ICH injury.

In the mitochondrial respiratory chain, CoQ10 acquires electrons from complex I, transports protons through the inner mitochondrial membrane, and subsequently transfers the electrons to complex II. Furthermore, it facilitates ATP production, where it serves as a cofactor in a set of redox reactions associated with ATP synthesis in the electron transport chain [27]. CoQ10 deficiency is related to most neurodegenerative diseases like Alzheimer’s, Huntington’s, as well as Parkinson’s diseases [28–30]. It can also stabilize membranes [31]. Several investigations have indicated that the drug may benefit patients with cardiovascular, neuromuscular, and neurodegenerative illnesses [31]. However, its effects on brain hemorrhage remains unclear [32]. CoQ10 deficiency in ICH can result in neuronal mitochondrial dysfunction and dysregulation of complexes I–III, which further leads to free radical production and oxidative stress [33], cell membrane injury caused by glutamate excitotoxicity [34], elevations of edema and blood–brain barrier permeability [35], neuroinflammation caused by glial cell overactivation [36], and neuronal apoptosis [37]. Therefore, we specifically assessed the impacts of CoQ10 on neuroinflammation and neuronal apoptosis in mouse brain tissue after ICH induction. Our data showed that a high dose of CoQ10 could alleviate neuroinflammation.

Inflammatory responses can worsen brain injury, which typically follows an ICH event. Pro-inflammatory reactions result in tissue injury, secondary edema, and eventual death of brain cells [24]. Inflammatory activation triggers hematoma enlargement, edema, and secondary neurological injury [25]. We provide the first evidence that CoQ10 can decrease pro-inflammatory cytokine levels 1 day after ICH, thereby protecting neurons from ICH-induced impairment. This research is also the first to indicate that the neuroprotective role of CoQ10 is mediated by anti-inflammatory reactions.

Given that apoptotic cell death is a dominant characteristic of neurotoxicity in perihematomal areas of the brain, inhibiting apoptosis would be a key strategy for reducing tissue damage and brain edema and improving functional outcomes in ICH [38, 39]. Nevertheless, no existing reports have explored the impacts of forkhead box protein M1 (*FoxM1*) gene knockdown or silencing on apoptosis during ICH. In the present research, TUNEL staining *in vivo* and flow cytometry *in vitro* were utilized to determine apoptotic cell death in the absence of FoxM1. The findings revealed marked decreases in the density of apoptotic cells in *FoxM1*-knockdown mice after ICH induction and in the proportion of apoptotic cells in *FoxM1*-silenced PCNs. Our data also indicated that *FoxM1* depletion attenuated apoptosis via the upregulation of Bcl-2 and the downregulation of Bax expression. Both proteins are executive molecules in the common apoptotic pathway, playing essential roles in regulating both caspase-dependent and -independent apoptosis. Collectively, the findings demonstrate that FoxM1 plays an anti-apoptotic role by elevating Bcl-2 and decreasing Bax expression in the ICH model.

To sum up, our research verified the function of CoQ10 in protecting against ICH-induced brain injury and that its neuroprotective effect is in part due to its inhibition of pro-inflammatory cytokine secretion and neuronal death. Furthermore, we confirmed that the inhibition of pro-inflammatory cytokine secretion by CoQ10 was modulated through its inhibition of p65 phosphorylation.

## Data Availability

The raw data supporting the conclusions of this article will be made available by the authors, without undue reservation.
