# Supplementary figures and images for: Coenzyme Q10 alleviates neurological deficits in a mouse model of intracerebral hemorrhage by reducing inflammation and apoptosis
Source: Exp Biol Med (Maywood). 2025 Feb 28;250:10321. doi: 10.3389/ebm.2025.10321 (PMC11906280; doi:10.3389/ebm.2025.10321)

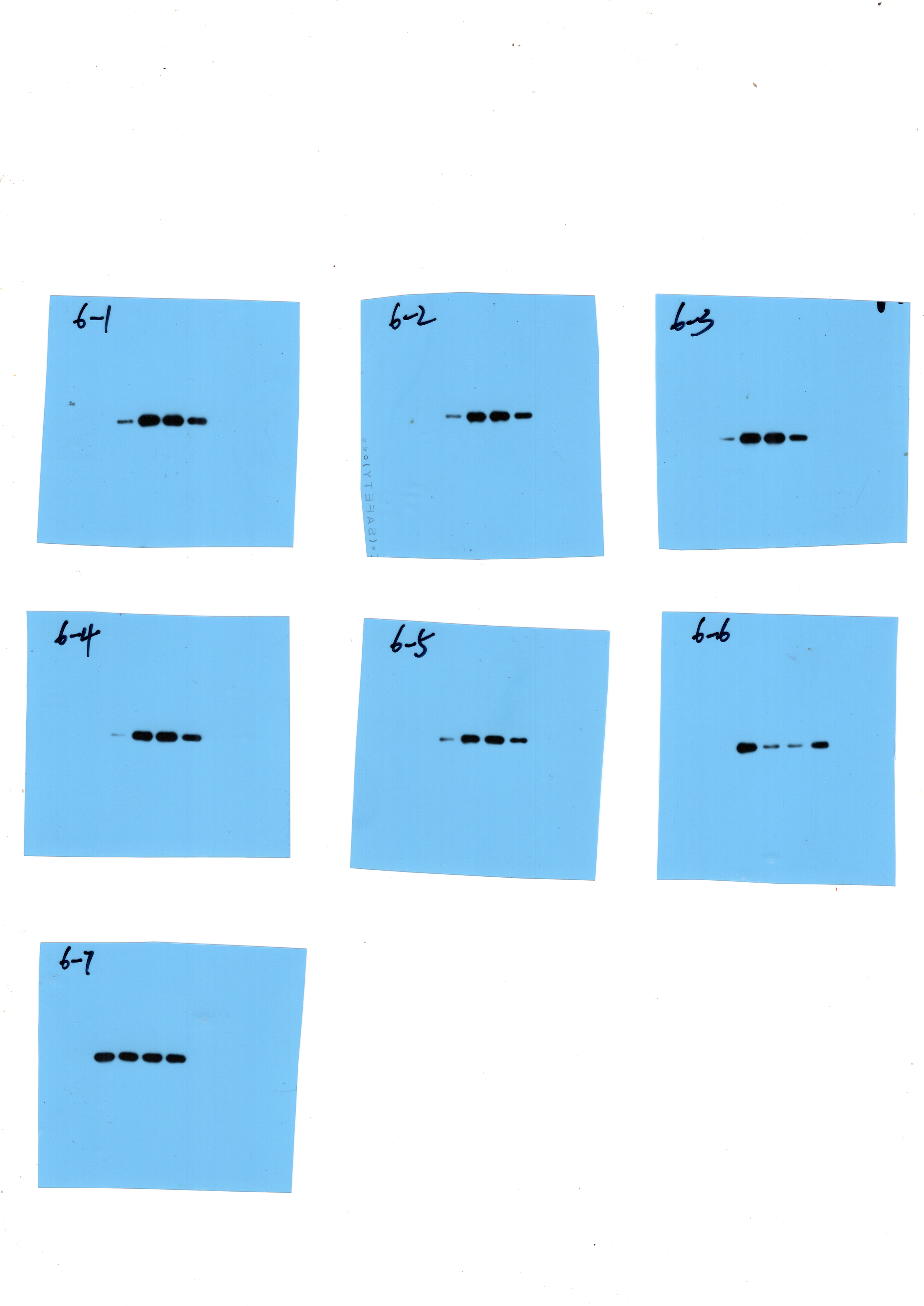

Supplement: Supplementary file 1 [file DataSheet1.ZIP › Raw WB images/Raw WB for figure 4.tif]

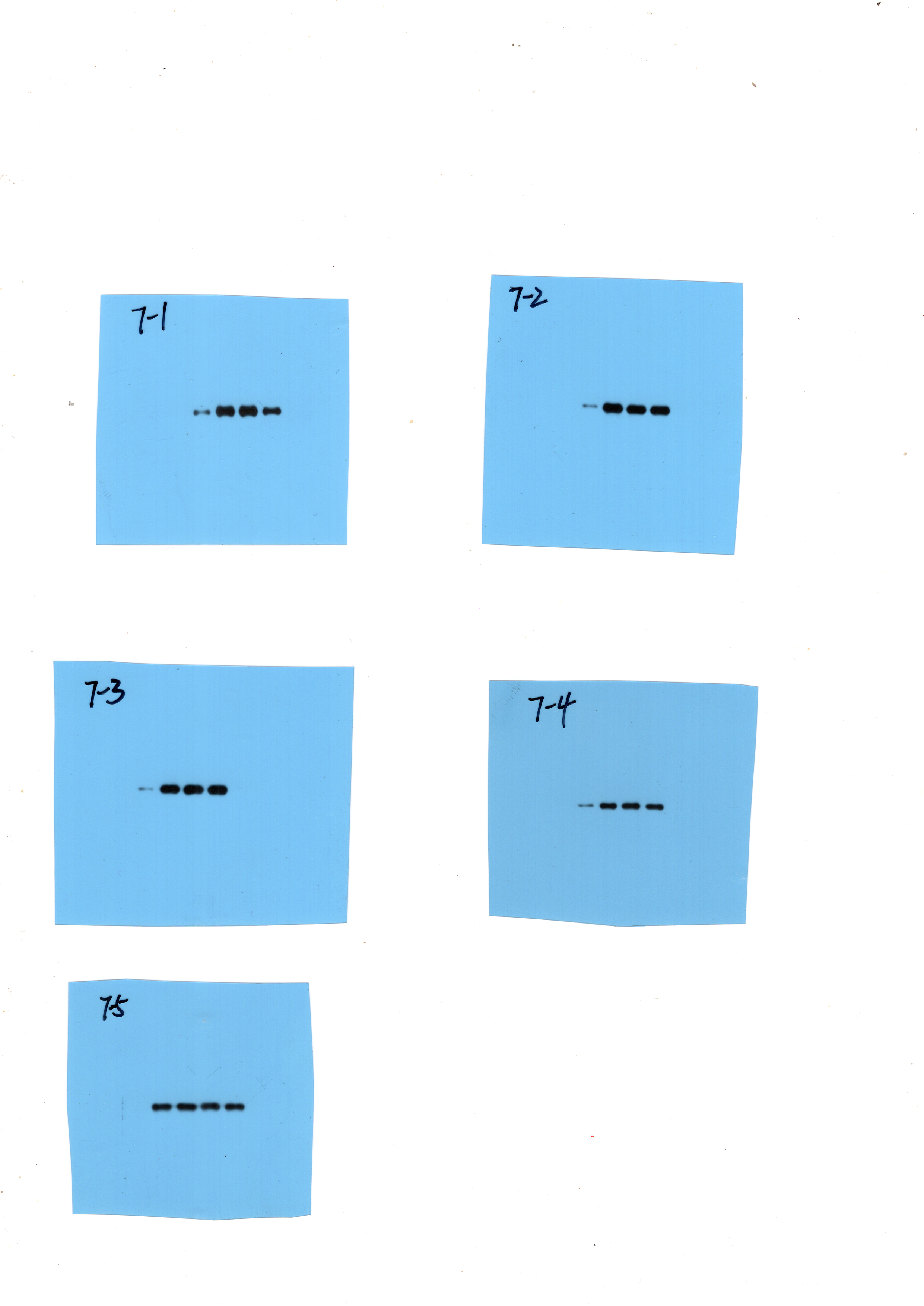

Supplement: Supplementary file 1 [file DataSheet1.ZIP › Raw WB images/Raw WB for figure 5.tif]
